# Rift Valley fever virus modulates apoptosis and immune response during infection of human astrocytes

**DOI:** 10.1080/22221751.2023.2207672

**Published:** 2023-06-22

**Authors:** Jordan Quellec, Aurélie Pédarrieu, Camille Piro-Mégy, Jonathan Barthelemy, Yannick Simonin, Sara Salinas, Catherine Cêtre-Sossah

**Affiliations:** a Pathogenesis and Control of Chronic and Emerging Infections, University of Montpellier, INSERM, Etablissement Français du Sang, Montpellier, France; b ASTRE, University of Montpellier, CIRAD, INRAe, Montpellier, France

**Keywords:** Rift Valley fever virus, arbovirus, astrocytes, apoptosis, immune response, interferon, neurovirulence

## Abstract

Rift Valley fever (RVF) is an arboviral disease of zoonotic origin that causes recurrent epidemics in Africa, the Arabic Peninsula, and islands of the South West of the Indian Ocean. RVF occurs mainly in livestock but also affects humans with severe clinical manifestations, including neurological disorders. However, human neuropathogenesis of Rift Valley fever virus (RVFV) is still poorly characterized. To study the interactions between RVFV and the central nervous system (CNS), we focused on RVFV infection of astrocytes, the major glial cells of the CNS that have several supporting roles including immune response regulation. We confirmed the permissiveness of astrocytes to RVFV infection and highlighted a strain-dependent infectivity. We showed that RVFV infection of astrocytes induced cell apoptosis and observed that the RVFV Non-Structural protein NSs, a known virulence factor, potentially delayed apoptosis by sequestrating activated-caspase 3 in the nucleus. Our study also showed that RVFV-infected astrocytes upregulated expression of genes associated with inflammatory and type I interferon responses at the mRNA level, but not at the protein level. This inhibition of immune response is potentially due to a NSs-dependent mechanism of mRNA nuclear export inhibition. Together, these results highlighted the direct impact of RVFV infection on the human CNS through the induction of apoptosis and a possible inhibition of early-onset immune responses that are crucial for the host survival.

## Introduction

Several emerging diseases are caused by viruses of zoonotic origin, including arthropod-borne viruses (arboviruses), and represent a major public health threat for humans [1]. Increased human activities, global warming, and environmental changes are involved in pathogen emergence: in this context, it is crucial to better understand, treat, and prevent the arrival of vector-borne viral diseases by improving our knowledge on arbovirus–host interactions [1,2]. Furthermore, some arboviruses such as Japanese encephalitis virus (JEV) or West Nile virus (WNV) cause epidemics involving a large burden of neurologic disorders [3]. Rift Valley fever (RVF), described in 1930 in Kenya [4] and caused by the RVF virus (RVFV), a negative single-stranded RNA virus from *Phlebovirus* genus, is one of these arboviruses of greatest concern for human and animal health [5]. RVFV is still responsible for alarming, re-occurring, and ongoing epidemics in endemic countries in Africa, the South West of the Indian ocean region, and the Arabian peninsula [6]. The lack of specific antiviral and preventive treatment including human vaccine, the poorly characterized pathogenesis mechanisms, and the increased risk of emergence place RVF on the World Health Organization list of “Prioritizing diseases for research and development in emergency contexts.”

RVFV has a complex biological cycle divided in a maintenance cycle in wildlife that allows RVFV persistence in the environment up to a decade and a complex transmission cycle from vector populations (e.g. *Aedes and Culex sp*.) to livestock (sheep, goats, cattle and camels), causing high mortality in young animals and high number of abortions [7]. However, the main transmission route for human infection is from direct contact with aerosolized particles from infected animal tissues and fluids [7]. Transmission by mosquito bite to humans is still discussed and inter-human horizontal transmission is undescribed at this time [7,8].

Given the inter-individual and inter-epidemic variability in terms of severity of RVFV infection, human mortality rate varies from 0.5% to 2% but can reach higher rates (e.g. 28.2% in Tanzania in 2007) [7,9–11]. Severe clinical signs are described: acute hepatitis, haemorrhagic fever, ocular diseases, and neurologic disorders [9,10]. The prevalence of neurologic disorders varies, depending on the studies and epidemics, but represent approximatively <1% to 21% of symptomatic cases [9,12]. These neurological forms are characterized by an acute or delayed meningoencephalitis occurring one to four weeks post-infection leading up to 50% of mortality and potential definitive disorder like incontinence or hemiparesis [9,10]. Associated with severe neurological symptoms (e.g. convulsion, hallucination, or coma), brain damages are characterized by tissues necrosis with lymphocytes and macrophages infiltration, lymphocytic pleocytosis, and perivascular cuffing [9,10,13].

Given that RVFV infection of rodents, non-human primates (NHP), and humans induces neurological disorders, it seems that RVFV can reach the brain [9,14–16]. Nowadays, RVFV neurotropism and neurovirulence are demonstrated in the rodent model with an active replication of RVFV in the central nervous system (CNS) cells [15,17–19], and with an early induced immune response associated with host survival, notably type I interferon (IFN) [20]. Moreover, CNS infection also induces a late immune response associated with infiltration of immune cells (macrophages, neutrophils, and lymphocytes), required for viral clearance but possibly leading to brain tissue damage [21–25]. However, RVFV neuroinvasion, neurotropism, and neuropathogenesis mechanisms in the human CNS are still poorly characterized [16,26,27]. Precisely, the potential origin of neurological disorders is not elucidated and could be due either to the establishment of a dysregulated immune response detrimental to the CNS homeostasis, or direct viral-induced cell lysis, or, most probably, to both mechanisms.

In the CNS, glial cells display numerous supportive functions for neuronal activity and survival. Astrocytes, one of these glial cells, are the most numerous cells in the brain and play a role in CNS physiological and metabolism regulation (neurotransmitters level, cerebral blood flow, energy metabolism, etc.) including immune responses [28]. In order to characterize neuroinfection mechanisms of RVFV in humans, we studied the interactions between astrocytes and RVFV through an *in vitro* approach assessing the permissiveness of astrocytes, cell viability, and associated-immune responses using different RVFV strains. Role of RVFV Non-Structural protein NSs, a major virulence factor forming nuclear filament and implicated in host immune response inhibition, was also explored [29].

## Materials and methods

### Viral stocks and quantification

Independent stocks of two RVFV strains isolated from the field, the strain MRU25010-30 (Passage 1, Mauritania, 2010, KM210508) [30], and the strain Mayotte 2008/00099 (Passage 1, Mayotte, 2008, HE687302-HE687304) [31], as well as the naturally NSs-delated strain Clone 13 (Passage 3, Bangui) [32], a kind gift from M. Bouloy (Institut Pasteur, Paris), were produced using African green monkey kidney Vero cells. Virus-containing medium was harvested when the cytopathic effect exceeded 75% and used as virus stock. For each virus stock and each experiment, viral infectivity titre from supernatant was determined by limiting dilution on Vero cells (TCID50, Tissues Culture Infectious Dose 50%) and calculated with Spearman–Kärber method [33].

### Cell culture and viral infections

The African green monkey kidney Vero cells were grown at 37°C-5% CO_2_ in MEM medium supplemented with 2 mM L-glutamine (Minimal Essential Medium, Gibco, USA) and 10% decomplemented foetal bovine serum (FBS) (Corning, France). Human primary astrocytes (ScienCell™, HA, 1800, lot 25672, USA), were maintained on flask or plate coated with Poly-D-Lysine 0.01% (Gibco, France) according to manufacturer’s instructions and used for RVFV infection (Passage 2–6). Astrocytes were infected at a multiplicity of infection (MOI) of 0.1 in a 6-well (1.8–3 × 10^5^ cells per well) or 12-well format (10^5^ cells per well) plates. Inocula prepared by diluting virus from viral stock in the appropriate culture medium were added during 90 min. A mock control corresponding to the inoculum with medium only was included for each infection. At indicated time point post-infection, supernatants and cells were harvested and stored at −80°C.

### Indirect immunofluorescence assays

To test human astrocytes infectivity, monolayers of astrocytes were infected during 48 h on 12-well plates containing coverslips coated with Poly-D-Lysine 0.01%. Cells were fixed using a 4% paraformaldehyde (PFA) solution during 15 min at room temperature (RT) and were then stored at 4°C. Cells permeabilization (5 min with Triton X-100 0.1%) and saturation steps (30 min in Phosphate-Buffered Saline (PBS) 1×, FBS 10% and Bovine Serum Albumin (BSA) 2%) were done before labelling with primary antibodies during 90 min at RT or overnight at 4°C and secondary antibodies during 1 h at RT. For the susceptibility assay, a mouse monoclonal IgG1 anti-N RVFV was used, whereas for the apoptosis assay, rabbit IgG anti-cleaved caspase 3, a component of apoptosis pathway [34] and mouse ascitic polyclonal Ig anti-NSs [35] (kindly given by Dr M. Flamand (Institut Pasteur, France)) were used. Astrocytes pre-treated with 25 mM of H_2_O_2_ during 2 h were used as positive control for apoptotic-specific labelling [36]. Goat polyclonal IgG anti-GFAP (glial fibrillary acidic protein) antibody and DAPI were used for cytoskeleton of astrocytes and nuclear labelling, respectively. Antibodies, dilutions, and reagents are detailed on supplementary data, Table S1. Labelled coverslips were mounted on glass slides with mounting medium (Fluoroshield, Sigma-Aldrich, France) and imaged by inversed microscope AXIOVERT A1 (Zeiss, France) using Archimed 6.1.4 software and analysed with Image J Software bundled with 64-bit Java 1.8.0_172. Infected and apoptotic cells were quantified by counting ≥1000 cells per sample.

### Fluorescence-activated cell sorting (FACS) assays

To explore cell viability post-RVFV infection, monolayers of astrocytes were infected during indicated time points, or pre-treated with 25 mM of H_2_O_2_ during 2 h as positive control for apoptosis [36]. After infection, cells were trypsinized and then pelleted in PBS 1X supplemented with 10% FBS by centrifugation (RT, 250 × g, 10 min). Staining of the exposure of phosphatidylserine at the cell surface, described as a morphological cell change during apoptosis [34], was performed with Annexin V:FITC assay Kit (BioRad, ANNEX300F, France) following manufacturer’s instructions using a one-step labelling. Following staining, cells were fixed with PFA 2% (20 min, RT). Data were then acquired by FACS Canto II flow cytometer (BD Biosciences, France) and analysed by FlowJo v10.0.7 software.

### Gene expression analysis

To explore the immune response induced by astrocytes after 48 h of RVFV infection, total RNA was extracted according to manufacturer’s instructions (RNeasy mini kit, Qiagen, USA) and was treated for genomic DNA elimination using RNase-free DNase Set (Qiagen, USA). The quality and the concentration of the isolated total RNA from each sample were checked using the NanoDrop 2000 (Thermo Fisher Scientific, France). Immune response was then screening by a two-step RT-qPCR kit targeting 84 different genes (Human Antiviral Response, Qiagen, USA, supplementary data, Table S2) on LightCycler 480 equipment following manufacturer’s instructions (Roche, France). Cycle threshold (Ct) values of targeted genes were normalized using housekeeping genes included in the kit and fold-changes of transcripts level compared to those of uninfected condition were determined by online Qiagen Software (RT² Profiler PCR Data Analysis). If fold-change values were <1, online Qiagen Software converted value *X* with −1/*X*.

### Cytokine and chemokine quantification

To explore the immune response induced by astrocytes after 48 h of RVFV infection at the protein level, harvested supernatants were tested using three types of LEGENDPlex kits (Human Anti-Virus Response Panel, Human pro-inflammatory chemokines Panel 1 and Human Neuroinflammation Panel 1, Biolegend, USA, supplementary data, Table S3) according to manufacturer’s instructions. Fluorescent signals (APC, PE) were measured with flow cytometer Canto II (BD Biosciences, France) and data analysed using the online Biolegend software (LEGENDplexTM Data Analysis).

### Nuclear mRNA export assay

To characterize the role of NSs factor on nuclear mRNA export in astrocytes after 48 h of RVFV infection, cell fractionation was performed on mock- and RVFV-infected astrocytes to separate nuclear and cytoplasmic fractions using the NE-PER Nuclear and Cytoplasmic extraction Reagents (Thermo Scientific, USA) following the manufacturer’s instructions. Then, total RNA was extracted as previously described and retrotranscription was performed with oligo (dT)_18_ using the Revert Aid First Strand cDNA synthesis kit (Fischer Scientific, USA) according to manufacturer’s instructions. To quantify mRNA levels in each fraction, gene-specific qPCR was then realized using the LightCycler 480 SYBR Green I Master kit (Roche, Switzerland, supplementary data, Table S4).

### Interferon assay

To determine the role of IFN response involved in the RVFV replication, astrocytes were pre-treated with three human recombinant IFN before infection: IFNα2 (pbl assay science, USA), IFNβ and IFNγ (R&D systems, USA). For these assays, cells were pre-treated with 500 UI/mL of each of the three IFN diluted in culture medium or with control (medium only) during 6 h. After IFN treatment, cells were infected during 24 h, then cells and supernatants were harvested as previously described.

### Statistical analysis

Each infection was done at least, in triplicate for each condition and measured in duplicate for each sample. Each experiment corresponds at least to two independent infections. Depending on data distribution and the type of assays, ANOVA two-way, Student’s *t*-test, Mann–Whitney test or Spearman correlation (*p < .05, **p < .01, ***p < .001, ****p < .0001) were used to analyse unpaired data with GraphPad Prism 9.5.0 software. For RT² Profiler PCR Data Analysis, online Qiagen software was used and data were analysed with Student’s *t*-test.

## Results

### RVFV infects and replicates in human astrocytes

**Figure 1.**
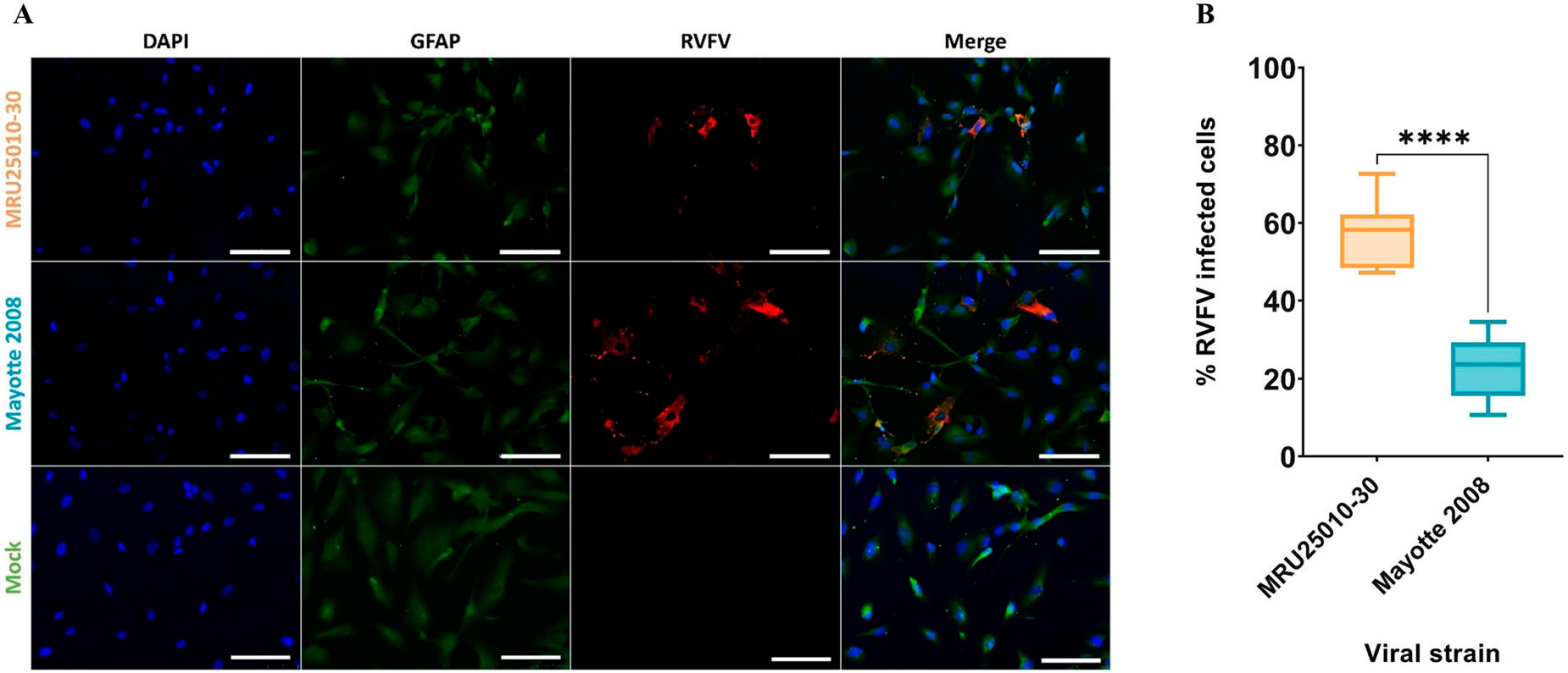
Human astrocytes are susceptible to RVFV infection. (A) Mock and RVFV-infected cells (MRU25010-30 and Mayotte 2008, MOI 0.1) were fixed at two days post-infection (dpi) and labelled with a monoclonal anti-N RVFV antibody (red), anti-GFAP antibody (green), and DAPI (blue) (scale bar 100 µm). (B) RVFV-infected cells (%) analysed by counting infected labelled cells compared to total cells number at 2 dpi (counting ≥1000 cells, *n* = 7, *t*-test: ****p-value < .0001).

**Figure 2.**
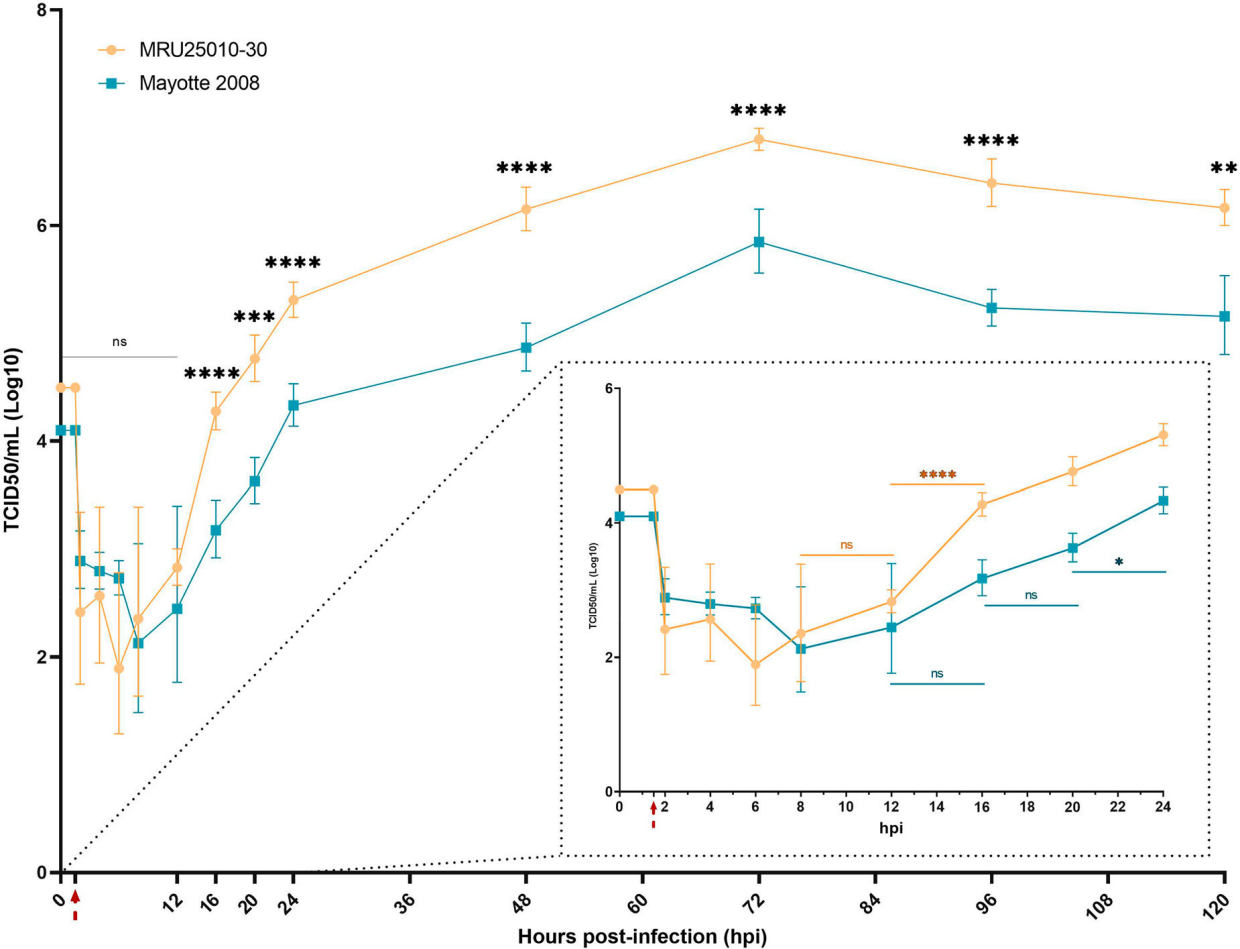
RVFV replicates in human astrocytes. Supernatants of mock- and RVFV-infected cells (MRU25010-30 and Mayotte 2008, MOI 0.1) were harvested at different time points post-infection and titrated using the TCDI50 method (Red arrow: removal of inoculum). Dotted square shows early infection time points. Results are expressed as geometric mean ±95% confidence interval (CI) and ANOVA two-way test show significance between each strain at each time point (black) or show significance between two time points for one strain (coloured, early times dotted square) (*p*-value: *p < .05, **p < .01, ***p < .001, ****p < .0001).

To evaluate the susceptibility of astrocytes to RVFV, primary human astrocytes were infected 48 h by RVFV strains Mayotte 2008 and MRU25010-30 and indirect immunofluorescence assays were performed on paraformaldehyde-fixed cells. We observed RVFV infection with the two RVFV strains using virus-specific labelling (antibody against the N nucleoprotein of RVFV) that was only present in infected astrocytes and absent in the mock-infected conditions (Figure 1(A)). However, we observed a significative strain-dependent variability at two days post-infection (dpi) in infection rates: MRU25010-30 infected 58.2% ± 8.74% of astrocytes while Mayotte 2008 infected 23.1% ± 7.82% (Figure 1(B)). We next determined RVFV replication rate by quantifying viral titres by TCID50 method in astrocyte supernatants harvested at different time points. This assay confirmed astrocyte permissiveness to RVFV: after 20 h post-infection (hpi), we showed a significant increase of viral titres for both strains, reaching a replication peak at 72 hpi (Figure 2). Furthermore, this assay confirmed the strain-dependent variability on infection rates. Indeed, MRU25010-30 displayed a significant replication between 12 and 16 hpi and Mayotte 2008 between 20 and 24 hpi. Similarly, MRU25010-30 had significant higher viral titres compared to Mayotte 2008 at each time point measured after 12 hpi.

Altogether, these results showed human astrocyte susceptibility and permissiveness to RVFV infection, but highlight a potential strain-dependent variability on infection efficiency.

### Infection of astrocytes by RVFV induces apoptosis but the RVFV NSs potentially delays cell death

**Figure 3.**
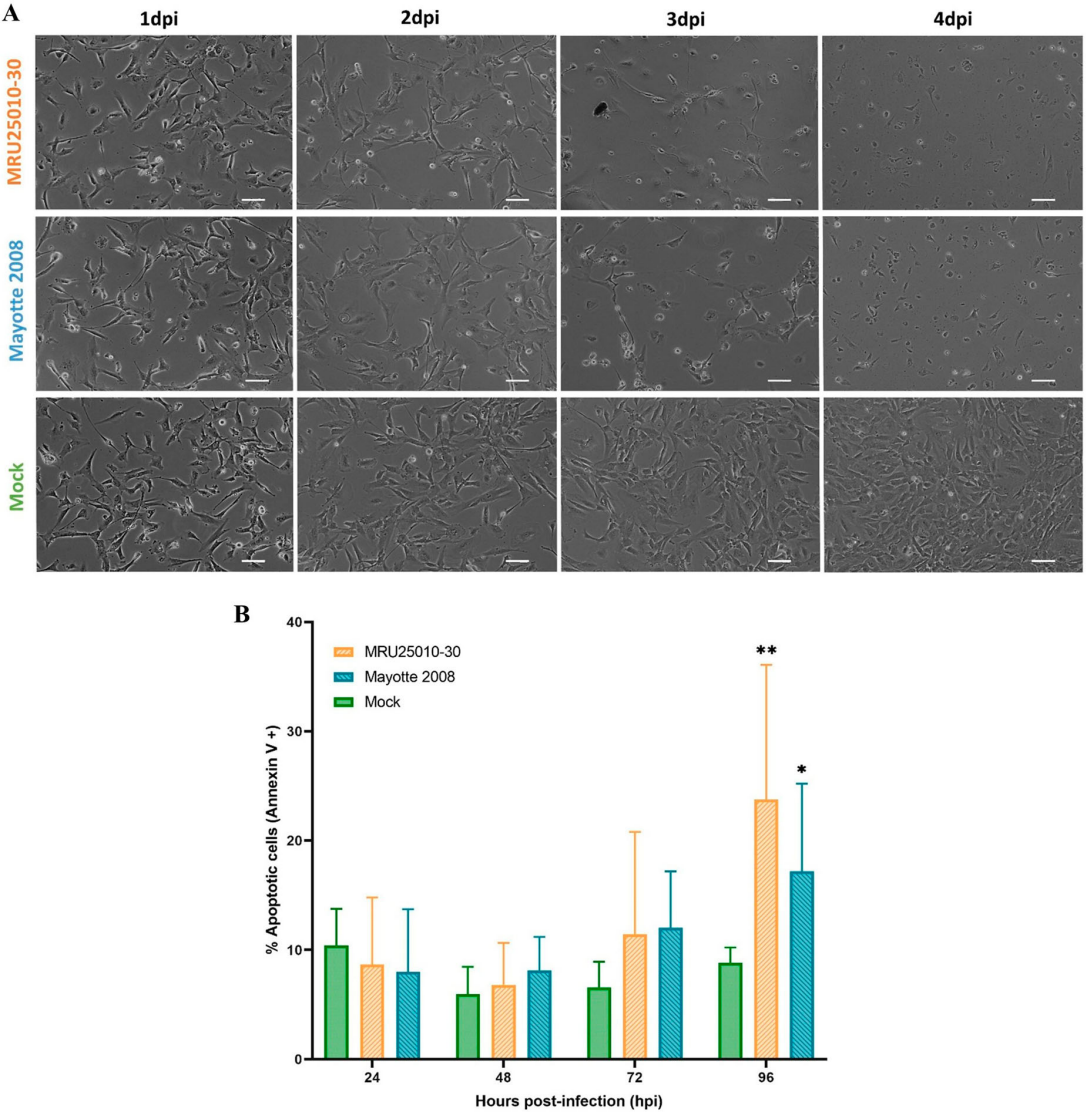
RVFV astrocyte infection induces early cell toxicity but a delayed apoptosis. (A) Cell morphology after RVFV infection analysed by phase contrast microscopy (scale bar 100 µm) at different days post-infection (dpi) in mock- and RVFV-infected cells (MRU25010-30 and Mayotte 2008, MOI 0.1). (B) Annexin V staining of mock and RVFV-infected cells (MRU25010-30 and Mayotte 2008, MOI 0.1) at different time points and measured by FACS. Results show percentage of cells positive for Annexin V expressed in mean ±95% confidence interval (Mann–Whitney: *p-value < .05, **p-value < .01).

Next, we monitored the effect of RVFV infection on astrocyte homeostasis. Indeed, we observed important morphological changes of infected cells between 48 and 72 hpi compared to uninfected cells, notably cell shrinkage and rounding as well as a potential decrease of cell proliferation, possibly due to infection-induced early cellular toxicity (Figure 3(A)). To explore the possible mechanisms involved in cell death, we quantified apoptosis at different time points post-infection using FACS analysis on astrocytes labelled with Annexin V, which has a strong affinity for phosphatidylserine, an early morphological change at cell surface during apoptosis. Using this approach, we showed that apoptosis was induced over time by the two RVFV strains (Figure 3(B)). Interestingly, astrocytes began to express significantly extracellular phosphatidylserine at a later stage (i.e. between 72 and 96 hpi), in approximatively 20% of cells, for both viral strains.

**Figure 4.**
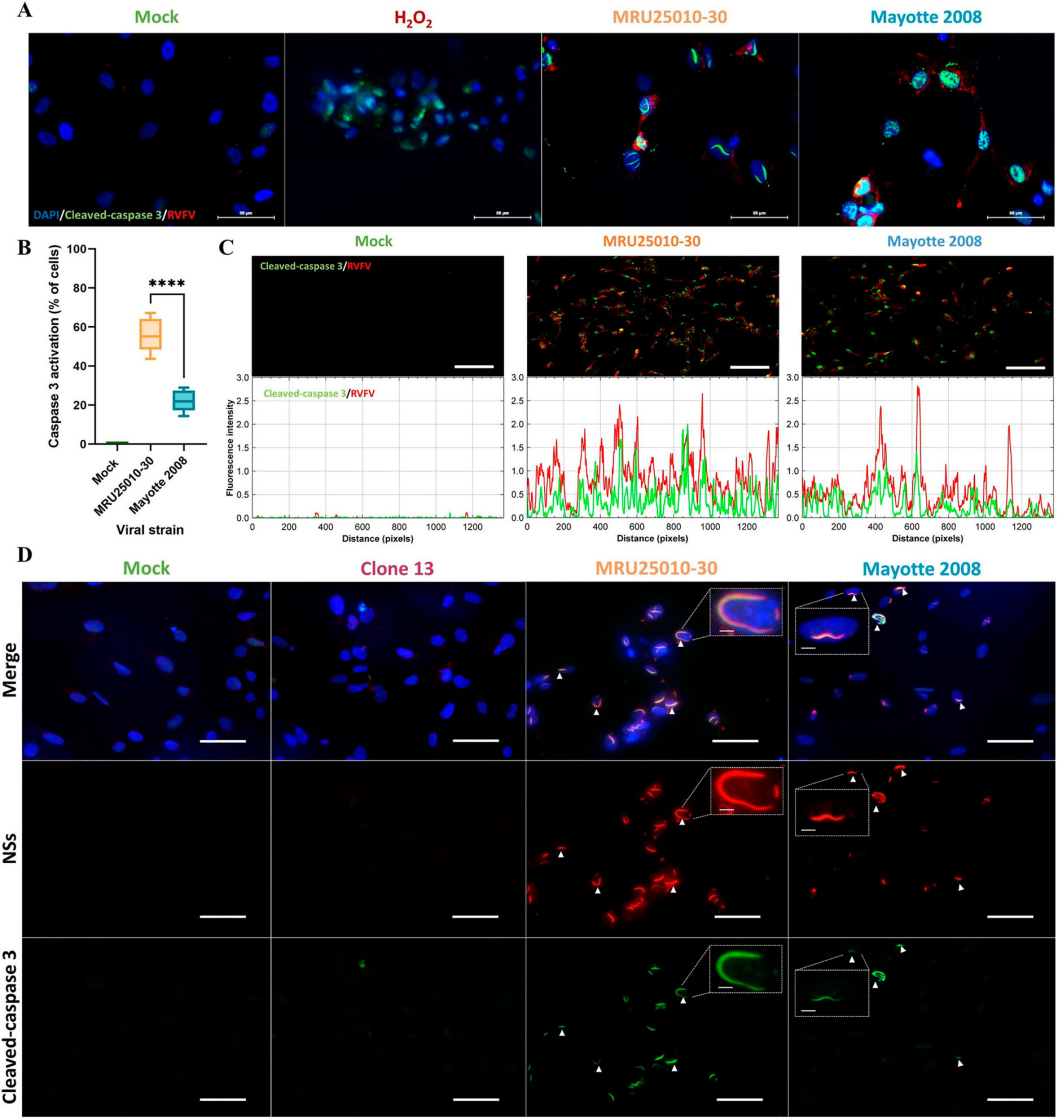
RVFV infection of astrocytes induces an early activation of caspase 3 and its potential intranuclear sequestration by NSs filaments. (A) Monitoring of cell apoptosis using mock-, H_2_O_2_ pre-treated – and RVFV-infected astrocytes (MRU25010-30 and Mayotte 2008, MOI 0.1) fixed at two days post-infection (dpi) and labelled with anti-cleaved caspase 3 antibody (green, Cas3), anti-RVFV antibody (red), and DAPI (blue) (scale bar 50 µm), (B) Quantification of apoptosis in RVFV-infected astrocytes (MRU25010-30 and Mayotte 2008, 2 dpi, MOI 0.1) by counting cleaved-caspase 3 labelled cells compared to total cell number (counting ≥ 1000 cells, *n* = 6, *t*-test: ****p-value < .0001). (C) Fluorescence intensity plots (bottom plots) measurement of indirect IFA pictures (top pictures, scale bar 200 µm) with anti-cleaved caspase antibody (green) and anti-RVFV antibody (red) in mock and infected conditions (MRU25010-30 and Mayotte 2008, 2 dpi, MOI 0.1). Intensity of fluorescence is calculated for each pixel in horizontal distance and for anti-RVFV signal (red) and anti-cleaved-caspase 3 signal (green). (D) RVFV NSs interaction with cleaved-caspase 3 was assay with mock and RVFV-infected astrocytes (MRU25010-30, Mayotte 2008 and Clone 13, MOI 0.1) fixed at 2 dpi. Cells were labelled with anti-NSs antibody (red), anti-cleaved caspase 3 antibody (green), and DAPI (blue) (scale bar 50 µm, small scale bar 4 µm). Yellow labelling corresponding to the colocalization of cleaved-caspase 3 and NSs (examples of colocalization by white arrows).

To further explore the importance of the apoptotic pathway in RVFV-induced astrocyte mortality, we performed an immunofluorescence assay to detect the pro-apoptotic molecule activated- (or cleaved-) caspase 3, which is an essential protease of many apoptotic pathways, after 48 hpi. First, we confirmed specificity of cleaved-caspase 3 labelling since labelling was absent in mock-treated cells and present in H_2_O_2_ pre-treated cells, a positive control known to induce apoptosis (Figure 4(A)). Using this approach, we confirmed a significant induction of caspase 3 cleavage in RVFV-infected cells (Figure 4(A,B)). Corroborating the infectivity variability previously described, apoptosis induced by RVFV infection was also strain-dependent: we observed more cleaved-caspase 3 positive cells in MRU25010-30-infected astrocytes than in Mayotte 2008-infected cells (Figure 4(B)). A Spearman correlation between the number of apoptotic cells (cleaved-caspase 3 positive) and the number of infected cells independent of the strain used (RVFV positive) showed a positive and significant correlation between these two labelling (*r* = 0.943, *p* < .0001, *n* = 4, data not shown). Furthermore, the comparison of the fluorescence intensity showed that RVFV and cleaved-caspase 3 levels appear to be visually correlated (Figure 4(C)). Regardless of experimental conditions (i.e. mock- or RVFV-infected), this positive and quantitative correlation was confirmed by Spearman correlation between fluorescence intensity values of RVFV and cleaved-caspase 3 staining (*r* = 0.76, *p*-value < .0001, *n* = 70, data not shown). These results suggest that astrocyte infection by RVFV induces directly caspase 3 cleavage and that caspase 3 activation increases along with RVFV replication.

Because activated-caspase 3 staining was detected at earlier time than phosphatidylserine exposure at cell surface, this led us to monitor the potential effect of RVFV infection in the modulation of the apoptosis pathway. Given this and the nuclear filament-like localization of cleaved caspase 3, we next explored the potential role of NSs, a major virulence factor of RVFV known to form nuclear filaments in infected cells, in apoptosis modulation. First, immunolabelling of NSs in infected astrocytes confirmed the nuclear NSs filament location during MRU25010-30 and Mayotte 2008 infection, as well as its absence during NSs-deleted RVFV strain Clone 13 infection (Figure 4(D)). However, we could highlight a strain-dependent kinetic of nuclear filament formation: NSs fibrilization is more important after 48 h of MRU25010-30 infection compared to Mayotte 2008 infection (Mann–Whitney, *p*-value < .0001, *n* = 45, data not shown). The co-staining of NSs and cleaved-caspase 3 in MRU25010-30 and Mayotte 2008 infected astrocytes showed a colocalization of these two proteins in the nucleus of infected-astrocytes at 48 hpi, suggesting of a potential retention of cleaved-caspase 3 in the nucleus by NSs (Figure 4(D)).

Altogether, these results suggest that astrocytes RVFV infection induces directly early cell toxicity and activation of the apoptosis pathway (cleaved-caspase 3) but apoptosis establishment and cell mortality seem to be delayed by a potential nuclear sequestration of activated-caspase 3 by RVFV NSs filament.

### RVFV astrocyte infection increases expression of genes related to interferon type I and inflammatory responses, without upregulation at the protein level by a potential nuclear sequestration of mRNA

**Figure 5.**
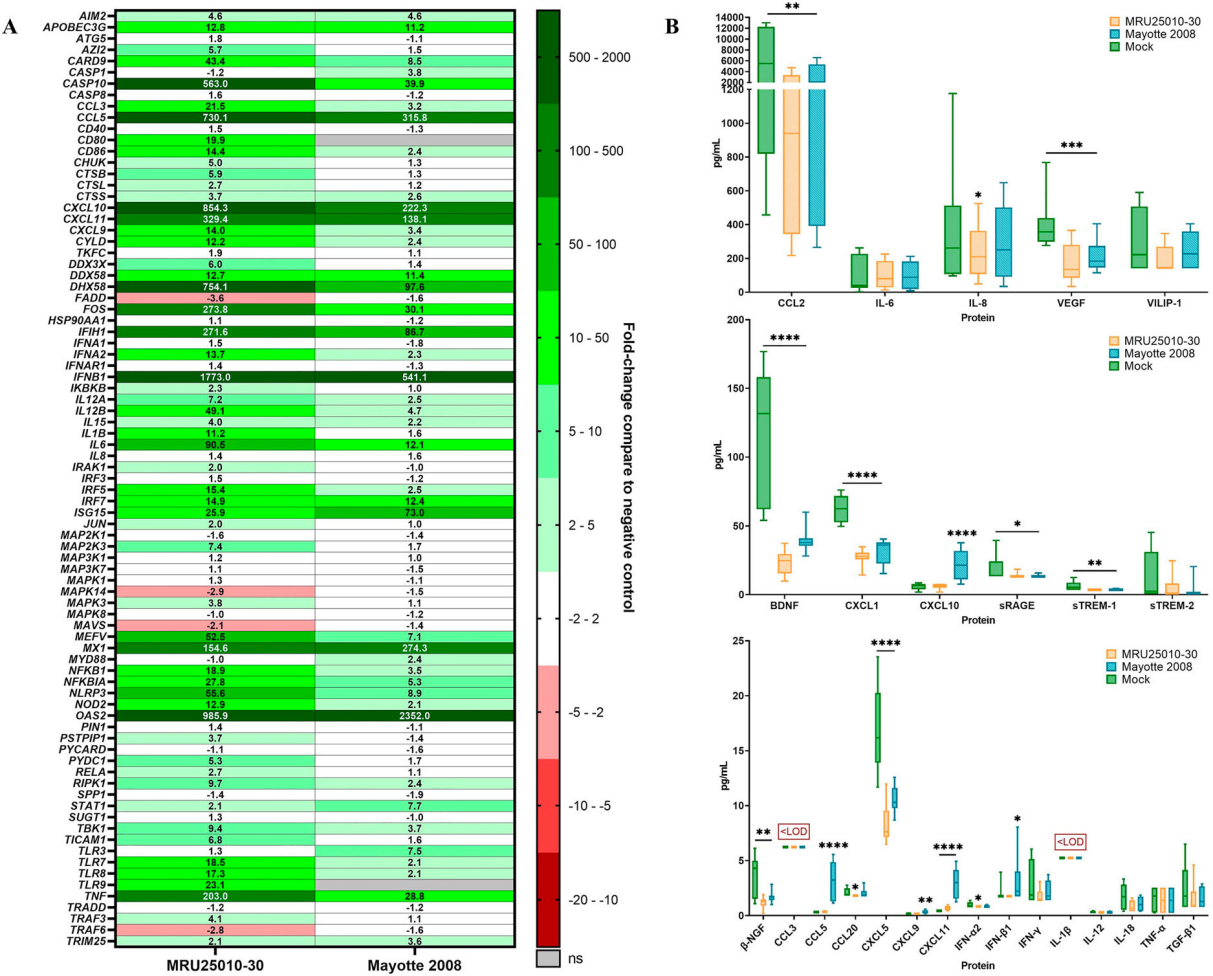
Astrocytes infection by RVFV induces inflammatory and antiviral responses at the mRNA level but not at the protein level. (A) Analyse of immune response induced by RVFV-infected astrocytes (MRU25010-30 and Mayotte 2008, MOI 0.1) after 48 h post-infection (hpi) assayed by measuring gene mRNA upregulated (green) or downregulated (red) expression with RT-qPCR using fold-change method standardized on housekeeping genes. All presented results have a *p*-value < .05 (*t*-test) or ns for non-significant. (B) Cytokines secreted in RVFV-infected astrocytes (MRU25010-30 and Mayotte 2008, MOI 0.1, 2 dpi) were measured using FACS multiplex assays (LOD: Limit of Detection) and compared to mock concentration (Mann–Whitney test compared to mock concentration, *p*-value: *p < .05, **p < .01, ***p < .001, ****p < .0001, with black bar for both strains, and without a bar when only one strain is considered).

To explore the immune response induced by RVFV-infected astrocytes after 48 h, we used a multi-array RT-qPCR assays. Independently of the viral strain, we showed an upregulation of numerous genes of the type I interferon (IFN) response: specific RNA virus pathogen recognition receptors (PRRs) components (*RLR: DDX58 (RIG-1), IFIH1/DHX58 (MDA5/LGP2)*), signalling pathway components (*IRF7*, *IRF5*, and *STAT1*), *IFNΒ1*, *IFNΑ2*, and antiviral effectors (*MX1*, *OAS2*, *ISG15*, and *APOBEC3G*) (Figure 5(A)). Furthermore, we detected an upregulation of others PRRs (*AIM2*, *NOD2*, *TLR7*, and *TLR8*), pro-inflammatory genes implicated in the cell inflammation signalling pathway (*NFKB*, *NFKBI (IKB)*, *TBK1*, *NRLP3*, and *TRIM25)* and pro-inflammatory cytokines and chemokines (*CCL3*, *CCL5*, *CXCL9*, *CXCL10*, *CXCL11*, *IL6*, *IL12*, *IL15*, *MEFV*, and *TNF*). Similarly, we showed an upregulation of genes implicated in the regulation of apoptosis (*CARD9*, *CASP10*, *FOS*, and *RIPK1*), which is consistent with previously observed apoptosis modulation, and other cell activities (*CTSS*, *CD86*, and *CYLD*). Furthermore, MRU25010-30 induced higher level of expression for these genes compared to Mayotte 2008, except for IFN effectors (*ISG15*, *MX1*, and *OAS2*), suggesting that Mayotte 2008 induces a more efficient IFN response. However, some genes were only overexpressed following MRU25010-30 infection, and notably genes encoding for others PRRs and components implicated in RNA virus recognition (*TLR9* and *TICAM1*), intracellular effectors and signals of inflammation (*AZI2*, *CHUK*, *CTSB*, *CTSL*, *DDX3X*, *IKBKB*, *IRAK1*, and *RELA*) and apoptosis (*JUN*, *TRAF3*, and *TRAF6*), as well as gene coding pro-inflammatory cytokines *IL1B*. Interestingly, only MRU25010-30 induced downregulation of *MAVS*, an important effector of IFN pathway. Mayotte 2008 infection also induced upregulation of specific genes such as *CAPS1*, *MYD88*, and *TLR3*.

We then investigated whether gene modulation was consistent with modulation at the protein level in supernatants of 48 h RVFV-infected cells compared to mock-infected conditions by FACS multiplex assay. Interestingly only Mayotte 2008 induced an increased secretion of IFN-β, CXCL9, CXCL10, and CCL5, and only CXCL11 was increased for both RVFV strains infection (Figure 5(B)). Furthermore, for the remaining analysed proteins and for both strains, several proteins had a concentration equivalent of the mock-infected condition (CCL3, IFN-γ, IL-1β, IL-6, IL-12, IL-18, sTREM-2, TNFα, TGF-β1, and VILIP-1) or had a lower concentration (BDNF, β-NGF, CCL2, CXCL1, CXCL5, sRAGE, sTREM-1, and VEGF).

**Figure 6.**
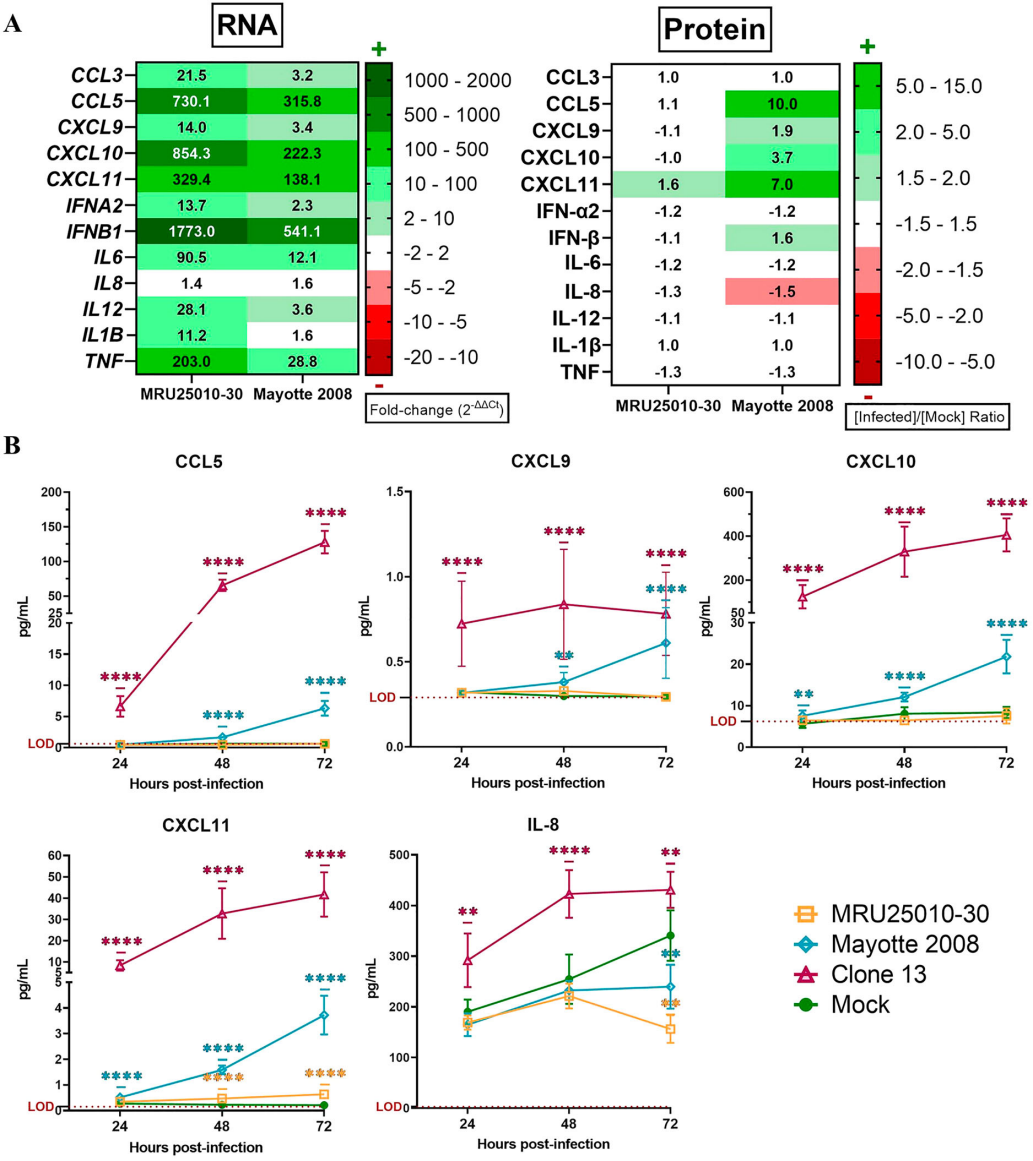
NSs potentially modulates astrocytes immune response by interfering with mRNA translation. (A) Comparison between mRNA and protein expression levels after 48 h post-infection (hpi) for specific genes: mRNA levels are expressed in fold-change and protein level with a ratio of mean protein concentration between infected and mock conditions. If protein ratio was under 1, value is expressed after −1/X conversion. (B) Kinetic of expression of inflammatory cytokines of RVFV-infected astrocytes (MRU25010-30, Mayotte 2008 and Clone 13, a NSs-delated natural strain, MOI 0.1). Measurements of protein concentration were done using FACS multiplex assays (LOD: Limit of Detection) at 24, 48, and 72 hpi. Results show protein concentration expressed in mean ±95% confidence interval (Mann–Whitney test compared to mock concentration, *p*-value: *p < .05, **p < .01, ***p < .001, ****p < .0001).

**Figure 7.**
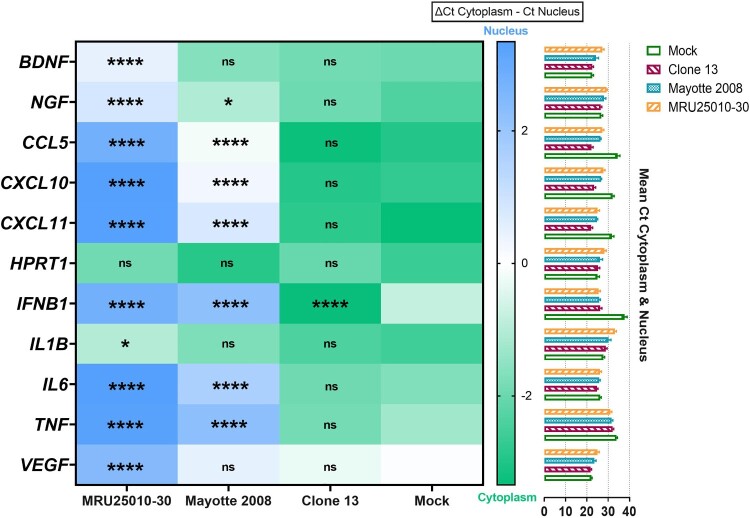
NSs potentially modulate immune response by inhibiting mRNA export from nucleus to cytoplasm. Relative quantities of mRNA related to housekeeping gene (*HPRT1*), to other cellular factors (*BDNF*, *NGF*, *VEGF*) or to immune response genes (*CCL5*, *CXCL10*, *CXCL11*, *IFNB1*, *IL6*, *IL1B*, *TNF*) were explored in the different cell compartments by RT-qPCR on RVFV-infected astrocytes (MRU25010-30, Mayotte 2008 and Clone 13, a NSs-delated natural strain, MOI 0.1). After 48 h of RVFV infection, cell fractionation was performed on astrocytes to get separate cytoplasm and nucleus components followed by total RNA extraction. Relative quantification of mRNA was performed by RT-qPCR: the mean of Ct values ±95% confidence interval from cytoplasm and nucleus at each condition is mentioned on the right side of the figure. To determine nuclear export of specific genes, a ΔCt ratio was calculated for each sample by subtracting the Ct value of cytoplasm by the Ct value of nucleus. Mean of this ΔCt ratio and statistical values is represented as a heatmap (ANOVA two-way, test compared to mock ΔCt ratio, *p*-value: ^ns^*p* > .05, *p < .05, ***p < .001, ****p < .0001).

At 48 hpi, when we compared mRNA upregulation level to protein concentration for analysed genes, we observed that MRU25010-30 infection did not induce an upregulation of protein despite a strong upregulation of their mRNA levels, except for CXCL11 (Figure 6(A)). However, Mayotte 2008 infection induced a lower upregulation of the same genes that MRU25010-30, but the secretion of some of these proteins was significantly increased (Figure 6(A)). Because these results suggested that RVFV could interfere with immune response mRNA translation or protein synthesis, we next monitored using FACS assays the kinetic of expression of pro-inflammatory cytokines (CCL5, CXCL9, CXCL10, CXCL11, and IL-8) at different time points after astrocyte infection by different RVFV strains (Figure 6(B)). MRU25010-30 only induced CXCL11 upregulation over time and a significant downregulation of IL8 at 72 hpi. Mayotte 2008 induced a significant upregulation of CCL5, CXCL9, CXCL10, and CXCL11 over time and also a downregulation of IL8 at 72 hpi. Interestingly, the NSs-deleted strain Clone 13 induced a higher and earlier upregulation of all tested cytokines at 24 hpi with an increase over time. Indeed, despite CXCL9, other tested cytokines had a significant higher level at each time point during Clone 13 infection and compared to MRU25010-30 and Mayotte 2008 conditions. To explore the role of the NSs virulent factor of RVFV on protein synthesis inhibition, a cell fractionation was performed on mock- and RVFV-infected astrocytes at 48 hpi to obtain cytoplasm and nucleus components separately. Specific mRNA was quantified on each cell fraction by RT-qPCR (Figure 7). Compared to ΔCt ratio (Ct of cytoplasmic fraction subtracted by Ct of nucleus fraction for each sample) of mock and Clone 13 controls, astrocytes infected with MRU25010-30 or Mayotte 2008 strains had a significantly higher proportion in the nucleus of mRNAs related to upregulated immune response genes (*IL6*, *CCL5*, *CXCL10, CXCL11*, *IFNB1*, and *TNF*) and to other cellular factors (*BDNF, NGF*, and *VEGF*). However, astrocytes infected by NSs-deleted Clone 13 strain induced a higher proportion of mRNA in the cytoplasm (e.g. *IFNB1*) or have a distribution of mRNA not significantly different of the mock condition. Interestingly and except for *HPRT1, IFNB1, IL1B*, and *TNF*, the proportion of nuclear mRNAs is higher during the infection with MRU25010-30 compared to the infection with Mayotte 2008 (ANOVA two-way on ΔCt ratio, *p* < .01), corroborating strain-dependent variability of previous results on inhibition of protein secretion. Altogether, these results suggest that RVFV induces a type I IFN and inflammatory response during astrocyte infection, but modulates mRNA translation possibly through the NSs virulence factor by a strain-dependent inhibition of mRNA export from nucleus, and thus inhibits potent immune response establishment.

### Interferon type I response inhibits RVFV replication

**Figure 8.**
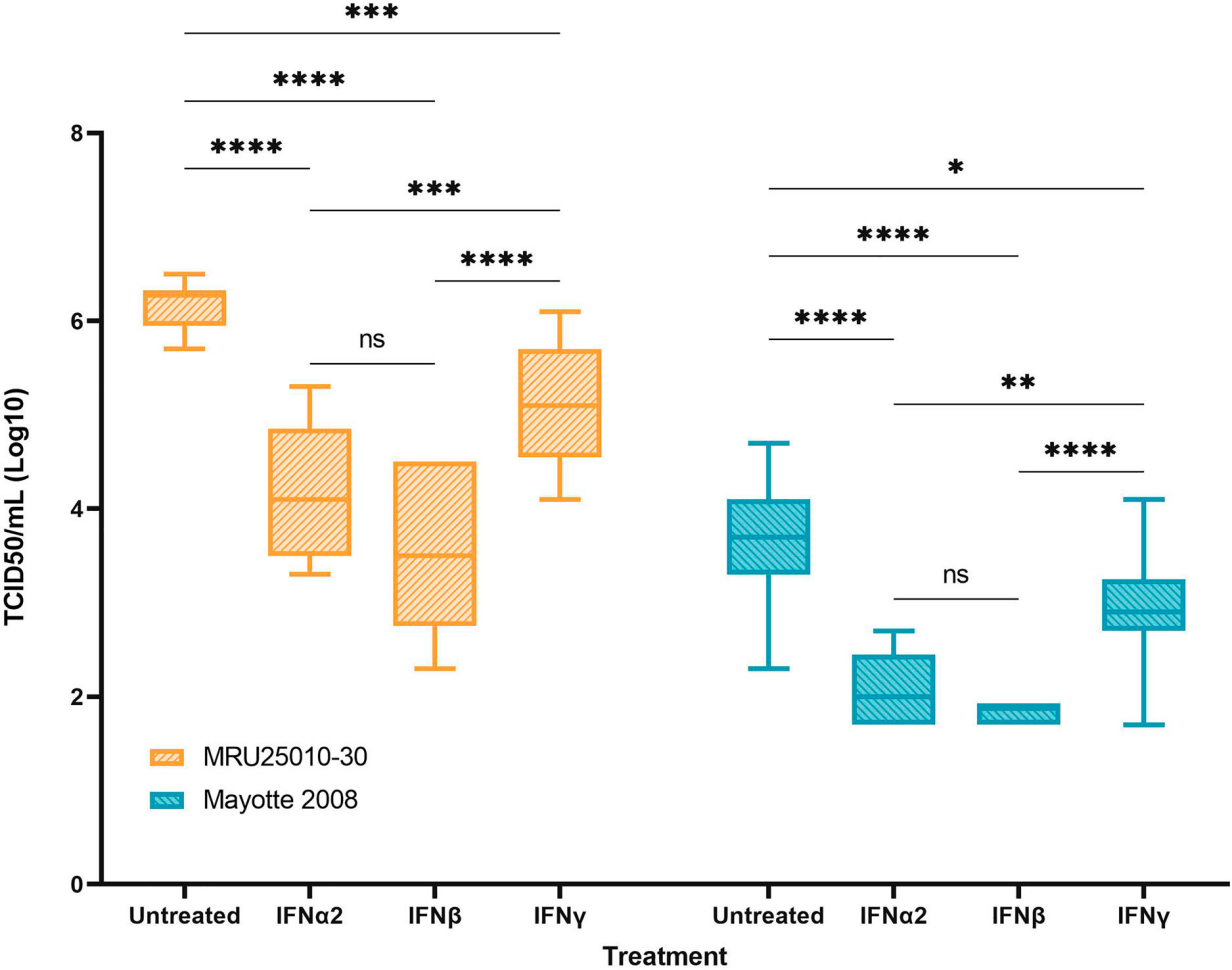
Type I and type II responses inhibit RVFV replication. In order to explore the role of the antiviral response induced by infected astrocytes on RVFV infection, RVFV titers of IFN pre-treated astrocytes (IFN-α2, IFN-β, and IFN-γ) are compared to pre-treated controls (medium only), 24 h post-infection with the two RVFV strains MRU25010-30 and Mayotte 2008. Viral titres were determined by TCID50 method (ANOVA two-way: *p*-value: *p < .05, **p < .01, ***p < .001, ****p < .0001).

We next explored the role of IFNs in RVFV infection of astrocytes. For both strains, we showed that pre-treatment of astrocytes with type I IFN (IFN-α2 and IFN-β) induced a potent significant inhibition of viral particles formation (Figure 8). Indeed, type I interferon response decreases viral titres by 2.3 log10 and 1.7 log10 for MRU25010-30 and Mayotte 2008, respectively. Furthermore, there was no significant difference between IFN-α2 and IFN-β inhibition. Inducing type II IFN response by IFN-γ pre-treatment also inhibited significantly viral replication for both strains, by 1 log10 and 0.7 log10 for MRU25010-30 and Mayotte 2008, respectively, but this inhibition was significantly lower than type I IFN response (Figure 8).

These results highlight the role of IFN responses, and notably type I IFN response, on RVFV replication inhibition by astrocytes and the importance for the virus to inhibit the IFN-related immune response to replicate efficiently. Type II interferon response also inhibited RVFV replication on astrocytes but at lower level.

## Discussion

Here, we demonstrated that RVFV was able to infect human astrocytes in vitro in a strain-dependent manner. Furthermore, we showed that RVFV infection induced astrocyte apoptosis by activation of caspase 3 with the involvement of NSs, one of the major virulence factor of RVFV, probably delaying induced-apoptosis by a potential sequestration of cleaved-caspase 3 in nuclear filaments. Moreover, during infection, astrocytes induced upregulation of immune response genes expression, and in particular a type I interferon response genes, which was shown to efficiently modulate RVFV replication. However, immune response at the protein level was not upregulated through a potential viral-mediated and strain-dependent inhibition of mRNA nuclear export.

Astrocytes play crucial and multiple supporting roles in CNS homeostasis. Indeed, astrocytes have regulating functions on neuronal signalling and metabolism, on blood–brain barrier (BBB) by regulating its integrity and permeability, and also on CNS immune system and viral clearance [28,37,38]. Thus, infection of astrocytes by RVFV can affect directly and indirectly CNS homeostasis and possibly leading to cognitive impairment. Indeed, the death of astrocytes leading to a loss of their supporting role, or the amplification of the neuroinflammation mediators, can lead to a decrease in neuron viability or BBB disruption, and therefore perturbation of the CNS homeostasis [37,38]. Given this perturbation, it was described that astrocytes functionalities impairment play a crucial role in the neuropathogenesis of infection and can lead to cognitive, behavioural, and motor disturbances [39,40].

In human RVFV infections, necrosis foci were described in deadly neurological forms, but the mechanistic causes of cell mortality are still poorly described in RVFV infection [26]. Induction of apoptosis was described, notably by TNF secretion and by DNA damage signalling caused by NSs nuclear filaments [16,41]. Here, we showed that astrocyte mortality during RVFV is potentially caused by an activation of intrinsic apoptosis pathway (caspase 3-dependent apoptosis), which is concordant with previous studies [42]. Furthermore, our data suggest that RVFV infection induces direct apoptosis of infected cells, as activation of the caspase 3 pathway is correlated with cell infection status, which is concomitant with the NSs-mediated DNA damage signal causing apoptosis.

Interestingly, the Non-Structural protein NSm is the virulent factor described in the anti-apoptotic activity by targeting protein at the mitochondrial outer membrane [43], but our result suggests that astrocyte apoptosis is potentially delayed by an anti-apoptotic activity of NSs filaments, and notably by hijacking activated-caspase 3 pathway by its nuclear sequestration. Indeed, activated-caspase 3 is responsible of scramblase activation, an enzyme leading to phosphatidylserine exposure at the cell surface [34] and, despite an early activation of caspase 3, the proportion of cells with extracellular phosphatidylserine outer membrane exposition was lower and only at a later stage of infection. Moreover, we showed that NSs filaments and activated-caspase 3 are both located in the same nuclear filaments, suggesting a potential sequestration role of NSs. Similarly, a recent report showed colocalization of NSs and activated-caspase 3 in the liver of RVFV-infected mice [44]. However, it will be interesting to explore molecular mechanisms allowing interactions between NSs and activated-caspase 3, using a quantitative evaluation of this specific function of NSs on viral replication.

Here, analyses of upregulated mRNAs during astrocytes infection showed a potent activation of RLR, type I IFN response and pro-inflammatory responses. These results are consistent with a ssRNA virus infection and following MAVS activation, which was already described as an important mediator to the establishment of a protective immune response in the brain of mice during RVFV infection [18,45,46]. Interestingly, we showed a potent upregulation of *IFNB1* mRNA, which was described as inhibited during RVFV infection [29,46]. Indeed, it was already described that NSs plays a crucial role in anti-type I IFN (IFN-α and IFN- β) activity, and notably by interaction with SAP30, leading to the suppression of IFN gene activation [29]. Furthermore, NSs anti-transcriptional activity on inducible and consistent general transcription was also described, and notably by its interaction with the transcriptional factor II H (TFIIH) [47]. In contrast, our results highlight a strong upregulation of genes associated with type I IFN, pro-inflammatory component and pro-apoptotic responses. This upregulation was already described in mouse models during RVFV infection of the liver, but at lower level in the spleen, suggesting an organ-dependent variability of transcriptional inhibition [48]. Whether this is due to the viral strain or the cell type studied remains to be determined.

Nevertheless, we showed an inhibition of the immune responses at translational level. Indeed, despite a potent upregulation of mRNAs, RVFV infection did not lead, and notably for the strain MRU25010-30, to an increase of secreted proteins concentration in cell supernatants. Moreover, for both strains, some cellular factors such as BDNF or β-NGF have a decreased concentration in infected conditions, suggesting that general cellular translation is potentially affected. To confirm these observations, it would be interesting to quantify the intracellular proteins which mRNAs were shown to be upregulated, such as *OAS2* or *RLR*, or enlarge studied proteins. Here, we showed that NSs was able to form nuclear filaments in astrocytes, filaments that were described as inhibitors of mRNA nuclear export [49], and thus possibly inhibiting translation of mRNAs and protein synthesis. Corroborating with this hypothesis, we showed that astrocytes infection by a NSs-deleted strain (Clone 13) induced a potent secretion of cytokines and chemokines that are limited or totally inhibited in Mayotte 2008 and MRU25010-30 infection. Furthermore, and corroborating with literature, our results confirm this hypothesis and highlight a mechanism of inhibition of mRNA nuclear export in astrocytes infected by MRU25010-30 and Mayotte 2008. Moreover, this mechanism seems strain-dependent and then possibly explains the strain-dependent infectivity of the RVFV strains used in this study. Thus, our results suggest that RVFV NSs play potentially an important role in inhibiting astrocyte protein synthesis by blocking mRNA nuclear export and thus inhibiting immune response establishment.

Furthermore, it seems crucial for RVFV to inhibit the viral-specific immune response, and notably IFN type I. Indeed, it was described that IFN type I, lead to an inhibition of RVFV genome replication at the early phase of infection, which is consistent with our results, and immune response lead to viral clearance at later stage by adaptative immunity activation [25,46]. Thus, variability on NSs anti-immune activity could correlate with replication variability of RVFV strains. Indeed, we showed that the higher virulent strain had potentially a faster NSs nuclear filament formation [50], inhibited more efficiently immune response and replicated earlier and at higher viral titres. In agreement with this hypothesis, our data showed that infection by the less virulent strain with slower NSs fibrilization induces a higher upregulation of IFN response effectors, a lowest inhibition of mRNA nuclear export and a significant secretion of IFN-β in astrocytes supernatant, suggesting a more effective IFN response of astrocytes.

During RVFV epidemics, human manifestations can harbour an important inter-individual variability in terms of severity and correlate with hosts immune response [51]. This crucial role of early innate immune response, and notably type I, was also described in mice and NHP [20,52]. Thus, variability of the severity of neurological forms of RVF could be explained by the strain variability of anti-immune activity of NSs. Indeed, NSs filament formation in infected cells was described as a crucial factor for neurovirulence in the mouse model [50]. Firstly, this inhibition promotes viral replication and it was described that viral titres positively correlate with mortality during RVFV infection [53,54]. Moreover, in humans, inhibition or dysregulation of the immune response induced by RVFV was already described [53,55], but the role of this response in RVFV pathogenesis is still discussed [26]. In the rodent model, RVFV dysregulation of the immune response coincides with tissue damage, late BBB disruption, potent recruitment of inflammatory cells, and adaptative immune cells to the brain, but the direct impact of dysregulated immune response on tissue damage is still unanswered [21–24]. Our study showed this immune dysregulation at an early stage of human glial cell infection, allowing RVFV replication and cell death by apoptosis, and dysregulation of immune regulation of adaptative response, but does not allow us to conclude about the consequences of this dysregulation at later stages of infection.

To conclude, our study showed a potent and strain-dependent impairment of human astrocytes during RVFV infection as well as a strain-dependent inhibition of the induced immune response, thus potentially promoting viral replication. Neurological disorders could be caused partially by infection of astrocytes and then indirectly by the early dysregulation of CNS homeostasis. Moreover, origin of strain-dependent variability and the implication of a dysregulated immune response on later nervous tissue damage are still uncertain and remained to be explored.

## Supplementary Material

Supplemental Material
