# Supplementary material for: Rift Valley fever virus modulates apoptosis and immune response during infection of human astrocytes
Source: Emerg Microbes Infect. 2023 Jun 22;12(1):2207672. doi: 10.1080/22221751.2023.2207672 (PMC10288929; doi:10.1080/22221751.2023.2207672)
Supplement: Supplemental Material [file TEMI_A_2207672_SM2950.docx]

# Appendices

Table 1: List of antibodies and reagents used in this study

| Target or product | Primary labeling antibody | Dilution or concentration (Reference) | Secondary labeling antibody | Dilution (Reference) |
| --- | --- | --- | --- | --- |
| RVFV N structural protein | Monoclonal mouse IgG1 anti-N RVFV | 1:50 (Cirad, 15G6-4B8) | Polyclonal donkey IgG anti-mouse IgG 555 | 1:500 (Invitrogen A31570) |
| Astrocyte GFAP | Polyclonal goat IgG anti-GFAP | 1:100 (Abcam ab53554) | Polyclonal rabbit Ig anti-goat Ig 488 | 1:50 (DAKO, F0250) |
| RVFV NSs protein | Polyclonal mouse Ig anti-NSs | 1:200 (Yadani et al., 1999) | Polyclonal donkey IgG anti-rabbit IgG 488 | 1:500 (Invitrogen A32790) |
| Cleaved-caspase 3 | Polyclonal rabbit IgG anti-cleaved Caspase 3 | 1:50 (Merck, AB3623) | Polyclonal donkey IgG anti-rabbit IgG 488 | 1:500 (Invitrogen A32790) |
| Cleaved-caspase 3 | Polyclonal rabbit IgG anti-cleaved Caspase 3 | 1:200 (Cell Signaling, 9661) | Polyclonal donkey IgG anti-rabbit IgG 488 | 1:500 (Invitrogen A32790) |
| Nucleus |  |  | DAPI | 1:1000 (Sigma-Aldrich, MBD0015) |
| Phosphate-buffered saline (PBS) |  | 1X (Sigma-Aldrich, D8537) |  |  |
| Triton 100X |  | 0.1% (Sigma-Aldrich, 93443) |  |  |
| Paraformaldehyde (PFA) |  | 4% (Sigma-Aldrich, P6148) |  |  |
| Bovine serum albumin (BSA) |  | 2% (Sigma-Aldrich, A7906) |  |  |
| H_2_O_2_ |  | 25mM (Sigma-Aldrich, H1009) |  |  |

Table 2: List of genes analyzed with the RT² Human Antiviral Response kit (QIAGEN, USA)

| **Gene** | **Description** | **Gene** | **Description** | **Gene** | **Description** |
| --- | --- | --- | --- | --- | --- |
| *AIM2* | Absent In Melanoma 2 | *IFNA2* | Interferon, alpha 2 | *NFKBIA* | Nuclear Factor of Kappa light polypeptide gene enhancer in B-cells Inhibitor, alpha |
| *APOBEC3G* | Apolipoprotein B mRNA Editing enzyme, Catalytic polypeptide-like 3G | *IFNAR1* | Interferon (alpha, beta and omega) Receptor 1 | *NLRP3* | NLR family, pyrin domain containing 3 |
| *ATG5* | Autophagy Related 5 | *IFNB1* | Interferon, beta 1 | *NOD2* | Nucleotide-binding Oligomerization Domain containing 2 |
| *AZI2* | 5-Azacytidine Induced 2 | *IKBKB* | Inhibitor of Kappa light polypeptide gene enhancer in B-cells, Kinase Beta | *OAS2* | 2'-5'-Oligoadenylate Synthetase 2, 69/71kDa |
| *CARD9* | Caspase Recruitment Domain family, member 9 | *IL12A* | Interleukin 12A | *PIN1* | Peptidylprolyl cis/trans Isomerase, NIMA-interacting 1 |
| *CASP1* | Caspase 1 | *IL12B* | Interleukin 12B | *PSTPIP1* | Proline-Serine-Threonine Phosphatase Interacting Protein 1 |
| *CASP10* | Caspase 10 | *IL15* | Interleukin 15 | *PYCARD* | PYD and CARD domain containing |
| *CASP8* | Caspase 8 | *IL18* | Interleukin 18 | *PYDC1* | PYD (PYrin Domain) Containing 1 |
| *CCL3* | Chemokine (C-C motif) Ligand 3 | *IL1B* | Interleukin 1, beta | *RELA* | V-rel Reticuloendotheliosis viral oncogene homolog A (avian) |
| *CCL5* | Chemokine (C-C motif) Ligand 5 | *IL6* | Interleukin 6 | *RIPK1* | Receptor (TNFRSF)-Interacting serine-threonine Kinase 1 |
| *CD40* | CD40 molecule, TNF receptor superfamily member 5 | *IL8* | Interleukin 8 | *SPP1* | Secreted PhosphoProtein 1 |
| *CD80* | CD80 molecule | *IRAK1* | Interleukin-1 Receptor-Associated Kinase 1 | *STAT1* | Signal Transducer and Activator of Transcription 1, 91kDa |
| *CD86* | CD86 molecule | *IRF3* | Interferon Regulatory Factor 3 | *SUGT1* | SGT1, suppressor of G2 allele of SKP1 (*S. cerevisiae*) |
| *CHUK* | Conserved Helix-loop-helix Ubiquitous Kinase | *IRF5* | Interferon Regulatory Factor 5 | *TBK1* | TANK-Binding Kinase 1 |
| *CTSB* | Cathepsin B | *IRF7* | Interferon Regulatory Factor 7 | *TICAM1* | Toll-like receptor Adaptor Molecule 1 |
| *CTSL* | Cathepsin L1 | *ISG15* | Interferon Stimulated Gene 15 | *TLR3* | Toll-Like Receptor 3 |
| *CTSS* | Cathepsin S | *JUN* | Jun proto-oncogene | *TLR7* | Toll-Like Receptor 7 |
| *CXCL10* | Chemokine (C-X-C motif) Ligand 10 | *MAP2K1* | Mitogen-Activated Protein Kinase Kinase 1 | *TLR8* | Toll-Like Receptor 8 |
| *CXCL11* | Chemokine (C-X-C motif) Ligand 11 | *MAP2K3* | Mitogen-Activated Protein Kinase Kinase 3 | *TLR9* | Toll-Like Receptor 9 |
| *CXCL9* | Chemokine (C-X-C motif) Ligand 9 | *MAP3K1* | Mitogen-Activated Protein Kinase Kinase Kinase 1 | *TNF* | Tumor Necrosis Factor |
| *CYLD* | Cylindromatosis (turban tumor syndrome) | *MAP3K7* | Mitogen-Activated Protein Kinase Kinase Kinase 7 | *TRADD* | TNFRSF1A-Associated via Death Domain |
| *TKFC* | Dihydroxyacetone kinase 2 | *MAPK1* | Mitogen-Activated Protein Kinase 1 | *TRAF3* | TNF Receptor-Associated Factor 3 |
| *DDX3X* | DEAD (Asp-Glu-Ala-Asp) box polypeptide 3, X-linked | *MAPK14* | Mitogen-Activated Protein Kinase 14 | *TRAF6* | TNF Receptor-Associated Factor 6 |
| *DDX58* | DEAD (Asp-Glu-Ala-Asp) box polypeptide 58 | *MAPK3* | Mitogen-Activated Protein Kinase 3 | *TRIM25* | Tripartite Motif containing 25 |
| *DHX58* | DEXH (Asp-Glu-X-His) box polypeptide 58 | *MAPK8* | Mitogen-Activated Protein Kinase 8 | *ACTB* | Actin, beta |
| *FADD* | Fas (TNFRSF6)-Associated via Death Domain | *MAVS* | Mitochondrial Antiviral Signaling protein | *B2M* | Beta-2-Microglobulin |
| *FOS* | FBJ murine osteosarcoma viral oncogene homolog | *MEFV* | Mediterranean Fever | *GAPDH* | Glyceraldehyde-3-Phosphate Dehydrogenase |
| *HSP90AA1* | Heat Shock Protein 90kDa Alpha (cytosolic), class A member 1 | *MX1* | Myxovirus (influenza virus) resistance 1 | *HPRT1* | Hypoxanthine PhosphoRibosyl Transferase 1 |
| *IFIH1* | Interferon Induced with Helicase C domain 1 | *MYD88* | Myeloid Differentiation primary response gene (88) | *RPLP0* | Ribosomal Protein, Large, P0 |
| *IFNA1* | Interferon, alpha 1 | *NFKB1* | Nuclear Factor of Kappa light polypeptide gene enhancer in B-cells 1 |  |  |

Table 3: List of proteins analyzed using the LEGENDPlex kits (BioLegend, USA)

| **Bead ID** | **Human Proinflammatory Chemokines Panel 1**  No. 740985 | | **Human Neuroinflammation Panel 1**  No. 740796 | | **Human Anti-Virus Response Panel**  No. 740390 | |
| --- | --- | --- | --- | --- | --- | --- |
|  | Protein | Description | Protein | Description | Protein | Description |
| A4 | IL-8 | Interleukin 8 | VILIP-1 | Serum Visinin-Like Protein 1 | IL-1β | Interleukin 1 beta |
| A5 | CXCL10 | Chemokine (C-X-C motif) Ligand 10 | CCL2 | Chemokine (C-C motif) Ligand 2 | IL-6 | Interleukin 6 |
| A6 | CCL11/24/26* | Chemokine (C-C motif) Ligand 11/24/26 | sTREM-2 | soluble Triggering Receptor Expressed on Myeloid cells-2 | TNF-α | Tumor Necrosis Factor alpha |
| A7 | CCL17* | Chemokine (C-C motif) Ligand 17 | BDNF | Brain-Derived Neurotrophic Factor | CXCL10 | Chemokine (C-X-C motif) Ligand 10 |
| A8 | CCL2 | Chemokine (C-C motif) Ligand 2 | TGF-β1 (Free Active) | Transforming Growth Factor beta 1 | IFN-λ1* | Interferon lambda 1 |
| A10 | CCL5 | Chemokine (C-C motif) Ligand 5 | VEGF | Vascular Endothelial Growth Factor | IL-8 | Interleukin 8 |
| B2 | CCL3 | Chemokine (C-C motif) Ligand 3 | IL-6 | Interleukin 6 | IL12a/IL12b | Interleukin 12 (S.U. A and B) |
| B3 | CXCL9 | Chemokine (C-X-C motif) Ligand 9 | sTREM-1 | soluble Triggering Receptor Expressed on Myeloid cells-1 | IFN-α2 | Interferon alpha 2 |
| B4 | CXCL5 | Chemokine (C-X-C motif) Ligand 5 | β-NGF | beta Nerve Growth Factor | IFN-λ2/3* | Interferon lambda 2 and 3 |
| B5 | CCL20 | Chemokine (C-C motif) Ligand 20 | IL-18 | Interleukin 18 | GM-CSF | Granulocyte-Macrophage-Colony-Stimulating Factor |
| B6 | CXCL1 | Chemokine (C-X-C motif) Ligand 1 | TNF-α | Tumor Necrosis Factor alpha | IFN-β1 | Interferon beta 1 |
| B7 | CXCL11 | Chemokine (C-X-C motif) Ligand 11 | sRAGE | soluble Receptor for Advanced Glycation End products | IL-10* | Interleukin 10 |
| B9 | CCL4* | Chemokine (C-C motif) Ligand 4 | CX3CL1* | Chemokine (C-X3-C motif) Ligand 1 | IFN-γ | Interferon gamma |

*Not detected due to concentration under limit of detection in all tested conditions and/or not included in the RT² gene panel.

Table 4: List of primers and RTqPCR cycling conditions.

| **Target gene** | **Forward primer 5’–3’** | | | **Reverse primer 5’-3’** | | | | | **Reference** |
| --- | --- | --- | --- | --- | --- | --- | --- | --- | --- |
| *BDNF* | GGATGAGGACCAGAAAGT | | | AGCAGAAAGAGAAGAGGAG | | | | | [1] |
| *NGF* | CCATCCCATCTTCCACAG | | | CTCTCCCAACACCATCAC | | | | | [1] |
| *CCL5* | CTCATTGCTACTGCCCTCTGCGCTCCTGC | | | GCTCATCTCCAAAGAGTTGATGTACTC | | | | | [2] |
| *CXCL10* | TATTCCTGCAAGCCAATTTTGTC | | | TCTTGATC*GCCTTCGATTCTG | | | | | [3] |
| *CXCL11* | GACGCTGTCTTTGCATAGGC | | | GGATTTAGGCATCGTTGTCCTTT | | | | | [4] |
| *HPRT1* | AGCTTGCTGGTGAAAAGGAC | | | TTATAGTCAAGGGCATATCC | | | | | [5] |
| *IFNB1* | GTCTCCTCCAAATTGCTCTC | | | ACAGGAGCTTCTGACACTGA | | | | | [6] |
| *IL1B* | ATGATGGCTTATTACAGTGGCAA | | | GTCGGAGATTCGTAGCTGGA | | | | | [7] |
| *IL6* | CCAGGAGCCCAGCTATGAAC | | | CCCAGGGAGAAGGCAACTG | | | | | [8] |
| *TNF* | CAGCCTCTTCTCCTTCCTGAT | | | GCCAGAGGGCTGATTAGAGA | | | | | [9] |
| *VEGF* | TGCAGATTATGCGGATCAAACC | | | TGCATTCACATTTGTTGTGCTGTAG | | | | | [10] |
| **RTqPCR cycling conditions** | | | | | | | | | |
| **Kit** | | Cycling conditions | | | | | | | |
|  |  | Preincubation | cDNA synthesis | Amplification | | | Melting curve/Inactivation | | |
| RevertAid First Strand cDNA synthesis (Fischer Scientific, USA) | | 65°C, 5min | 42°C, 60min |  | | | 70°C, 5min | | |
| LightCycler 480 SYBR Green I Master (Roche, Switzerland) | | 95°C, 10min |  | 95°C, 15s | 60°C, 15s | 72°C, 25s | 95°C, 5s | 65°C to 97°C continuously | |

*G instead of C in the reference [3]

[1] Esmaeili A, Alifarja S, Nourbakhsh N, et al. Messenger RNA expression patterns of neurotrophins during transdifferentiation of stem cells from human-exfoliated deciduous teeth into neural-like cells. Avicenna J Med Biotechnol. 2014;6:21–26.

[2] Vaday GG, Peehl DM, Kadam PA, et al. Expression of CCL5 (RANTES) and CCR5 in prostate cancer. Prostate. 2006;66:124–134.

[3] Eichholz K, Mennechet FJD, Kremer EJ. Human Coagulation Factor X-Adenovirus Type 5 Complexes Poorly Stimulate an Innate Immune Response in Human Mononuclear Phagocytes. J Virol. 2015;89:2884–2891.

[4] Gao YJ, Liu DL, Li S, et al. Down-regulation of CXCL11 inhibits colorectal cancer cell growth and epithelial-mesenchymal transition. Onco Targets Ther. 2018;11:7333–7343.

[5] Siednienko J, Nowak J, Moynagh PN, et al. Nitric oxide affects IL-6 expression in human peripheral blood mononuclear cells involving cGMP-dependent modulation of NF-κB activity. Cytokine. 2011;54:282–288.

[6] Faure E, Poissy J, Goffard A, et al. Distinct immune response in two MERS-CoV-infected patients: Can we go from bench to bedside? PLoS One. 2014;9.

[7] Wang H, Yuan M, Wang S, et al. STAT3 Regulates the Type I IFN-mediated antiviral response by interfering with the nuclear entry of STAT1. Int J Mol Sci. 2019;20.

[8] Chen Y, Pawlikowska L, Yao JS, et al. Interleukin-6 involvement in brain arteriovenous malformations. Ann Neurol. 2006;59:72–80.

[9] Li Z, Chao TC, Chang KY, et al. The long noncoding RNA THRIL regulates TNFα expression through its interaction with hnRNPL. Proc Natl Acad Sci U S A. 2014;111:1002–1007.

[10] Amirchaghmaghi E, Rezaei A, Moini A, et al. Gene expression analysis of VEGF and its receptors and assessment of its serum level in unexplained recurrent spontaneous abortion. Cell J. 2015;16:538–545.
